# Association between time of exposure to screens and food consumption of children aged 2 to 9 years during the COVID-19 pandemic

**DOI:** 10.1590/1984-0462/2023/41/2021284

**Published:** 2022-09-09

**Authors:** Julia Theisen Sacramento, Carolina Sant Anna de Menezes, Mariana Dall‘Agnol Brandão, Mônica Cristina Broilo, Daniele Botelho Vinholes, Fabiana Viegas Raimundo

**Affiliations:** a Universidade Federal de Ciências da Saúde de Porto Alegre, Porto Alegre, RS, Brazil.

**Keywords:** Screen time, Food consumption, Child, COVID-19, Parents, Tempo de tela, Consumo alimentar, Criança, COVID-19, Pais

## Abstract

**Objective:**

To identify and map families’ time of exposure to screens during the COVID-19 pandemic and to associate it with the frequency of children’s food consumption.

**Methods:**

This is a cross-sectional study carried out with parents or guardians of children aged between 2 and 9 years through an online questionnaire. The participants answered questions about socioeconomic data, family’s screen habits, and children’s food consumption.

**Results:**

A total of 517 parents or guardians participated in the research. Children’s median age when first exposed to screens was 6 months (interquartile range: 1–12 months). The average number of hours that children and guardians/parents were exposed to electronic devices was 3.9±2.3 and 9.0±2.9 respectively. As for food consumption, 60.9% of the guardians/parents and 54.3% of the children had the habit of having meals in front of screens. In addition, the consumption of snacks outside mealtimes, while using screens, was frequent in both groups.

**Conclusions:**

Children aged 2 to 9 years are excessively exposed to screens and the consumption of meals or snacks while using the devices is frequent. Considering the current demands of the pandemic, the necessity of using electronic devices is understandable. However, the authors emphasize the importance to educate families regarding limiting the use of screens, especially during meals, and monitoring the content of activities with electronic devices, as this exposure can influence food consumption and affect children’s nutritional status and health.

## RESUMO

**Objetivo:**

Identificar e mapear o tempo de exposição das famílias às telas durante a pandemia de COVID-19 e associá-lo com a frequência de consumo alimentar das crianças.

**Métodos:**

Trata-se de um estudo transversal, realizado com pais ou responsáveis de crianças de dois a nove anos de idade por meio de um questionário *online*. Participantes responderam a perguntas sobre dados socioeconômicos, hábitos familiares de uso de telas e consumo alimentar das crianças.

**Resultados:**

Participaram da pesquisa 517 pais ou responsáveis. A mediana de idade em que as crianças foram expostas pela primeira vez às telas foi de seis meses (intervalo interquartil: 1–12 meses). A média de horas durante as quais as crianças e os responsáveis ficavam expostos aos aparelhos eletrônicos foi de 3,9±2,3 e 9,0±2,9, respectivamente. Sobre o consumo de alimentos, 60,9% dos responsáveis e 54,3% das crianças tinham o hábito de realizar refeições em frente às telas. Além disso, o consumo de petiscos fora do horário das refeições, durante o uso de telas, foi frequente em ambos os grupos.

**Conclusões:**

Crianças de dois a nove anos estão expostas excessivamente às telas e o consumo de refeições ou petiscos durante o uso dos aparelhos é frequente. Diante das demandas atuais da pandemia, entende-se a importância do uso dos eletrônicos, porém reforça-se a necessidade de educar as famílias em relação à importância de limitar o uso de telas, especialmente durante as refeições, e de monitorar o conteúdo das atividades com telas, pois essa exposição pode influenciar a alimentação e repercutir no estado nutricional e na saúde das crianças.

## INTRODUCTION

In December 2019, the World Health Organization (WHO) received information about the outbreak of a new respiratory disease. This disease, transmitted by the new coronavirus, was called Coronavirus Disease 2019 (COVID-19).^1^ Thereafter, several measures were taken to contain its propagation, the main one being social distancing.^2^ As a consequence, face-to-face school activities were suspended, in such a way that children were restricted to the home environment and, in parallel, most adults were remotely working, which generated changes not only in the family dynamics, but also in the diet.^3^

Among aspects of the home environment that have repercussions on the quality of children’s diet, the time of exposure to screens is highlighted, defined as the time in which children stand in front of mobile phones, tablets, video games, televisions, and other digital devices.^4,5^ Studies show that changes in the lifestyle of the population are related to the longer time spent in front of screens,^6,7^ a fact that has worsened with the pandemic.^8,9^ The habit of gathering family members around the table for having meals has been replaced by the habit of having them in front of screens, which causes lack of attention, interfering with physiological signs of hunger and satiety and which may lead to inadequate food choices.^10^ The consumption of unhealthy foods throughout the day is directly related to the number of hours in front of the television.^11^ This fact is due to the strong influence of the media on eating behaviors, as the industry invests in advertisements for hyperpalatable and high-calorie foods, which can impact the formation of the eating habits of children and adolescents.^12^

During the quarantine period, schools transitioned from face-to-face to online classes, a teaching model that requires students to constantly use screens for accessing their studies, causing children to spend most of the day involved with digital devices. In a research conducted in Turkey during quarantine on parents of children aged 6 to 13 years, 71.7% of the parents stated that their children’s screen time increased during this period.^8^

In addition, interactions between children, their caregivers, and the environment in which they are inserted influence their exposure time to screens. A study conducted in the United States of America on guardians of children and adolescents aged between 0 and 17 years showed that the time that parents spend in front of screens is directly correlated with their children’s screen time.^13^ These interactions also influence the formation of children’s eating behavior, as parents act as the main models, playing a fundamental role in the development of eating habits.^14,15^

Considering that the family environment influences children’s food consumption and that, in the pandemic period, family and food dynamics may be undergoing changes, the aim of this study was to identify families’ time of exposure to screens during the COVID-19 pandemic and to associate it with the food consumption of children aged 2 to 9 years.

## METHOD

This is a cross-sectional study conducted online during the COVID-19 pandemic from November to December 2020 with parents or guardians of children aged 2 to 9 years. The research was disseminated through social networks (Instagram, Facebook, and WhatsApp), using an invitation text with the link to the questionnaire and explaining the objectives and procedures of the research. The first stage of the investigation consisted of reading and agreeing with the aspects described in the Informed Consent Form, which was electronically signed. Subsequently, the questionnaires were made available with questions about socioeconomic and demographic data, family habits of screen use, and children’s food consumption. The inclusion criterion was being a parent or guardian of children aged 2 to 9 years.

The sample size calculation was performed in the *Winpepi* program and used as a basis the prevalence of 47% of consumption of ultra-processed foods estimated by the study of Sparrenberger et al.^16^ Sampling error of 5%, 95% confidence level, and 10% increase for possible losses were considered, resulting in a minimum sample of 421 participants. The present study complies with all the guidelines of Resolution No. 466/2012 of the National Health Council of the Brazilian Ministry of Health and was approved by the Ethics and Research Committee of the Universidade Federal de Ciências da Saúde de Porto Alegre (UFCSPA), under Opinion No. 4.247.334.

Socioeconomic and demographic variables were collected through questions regarding children, guardians/parents, and the residence. Considering that no standardized and validated questionnaires were found to evaluate the time of screen use by children, this variable was collected using an instrument written based on the recommendations of the Brazilian Society of Pediatrics^17^ and the American Academy of Pediatrics.^18^ The questions were related to the age at which the child was first exposed to screens, the average daily time spent in this activity, the type of electronic device most used, the habit of using these devices during meals, and the habit of snacking during the use of screens by the children and their guardians. Furthermore, questions related to changes in the pattern of exposure to screens resulting from the current period of social distancing and remote school activities were asked.

Data on children’s food consumption were collected through a structured questionnaire, adapted from the instrument *Marcadores Alimentares – SISVAN, 2008* [“Food Markers – SISVAN, 2008”] of the Food and Nutrition Surveillance System (*Sistema de Vigilância Alimentar e Nutricional* – SISVAN).^19^ This questionnaire aimed to investigate the usual consumption of fresh or minimally processed and ultra-processed foods by children. The questions verify the frequency of consumption of the following foods in the seven days prior to the research: raw salad; cooked vegetables; fresh fruit or fruit salad; beans; milk or yogurt; French fries, potato chips, or fried snacks; hamburger or processed meats; instant noodles, packet snacks, or salted biscuits.

Data were collected directly from the *Research Electronic Data Capture* (RedCap) software. The *Statistical Package for the Social Sciences* (SPSS) program, version 25.0, was used to perform the statistical analyses. Descriptive analysis was performed by means and standard deviation for continuous variables of symmetric or median distribution, and interquartile range for continuous variables of asymmetric distribution. Categorical variables were described by absolute and relative frequencies. The association between the time of exposure to screens and the frequency of food consumption was verified with the application of the analysis of variance test (ANOVA). The adopted significance level was 5%.

## RESULTS

The study included 517 parents or guardians of children aged between 2 and 9 years. Mothers were the family members who answered the most to the survey, totaling 89.6%. Regarding level of education, 85.1% of the participants had a college degree and 50.7% received between 3 and 9 minimum wages, considering a minimum wage of BRL 1,045.00, the value in force in the country during 2020. The children’s average age was 5.3±2.4 years. The other characteristics of the sample are described in Table 1.

**Table 1.** Socioeconomic and demographic characteristics of parents or guardians, children aged two to nine years, and their homes.

	n (%) or mean (SD)
Data on guardians/parents	
Sex	
Women	481 (93.0)
Age (years)	36.4 (6.9)
Occupation	
Has a professional activity	406 (78.5)
Level of education (years)	
≤8 years of formal education	77 (14.9)
9 or more years of formal education	440 (85.1)
Marital status	
Have a partner	439 (84.9)
Degree of kinship	
Mother	463 (89.6)
Father	37 (7.1)
Grandfather/grandmother	1 (0.2)
Others	16 (3.1)
Monthly family income (minimum wages*)	
Up to two	71 (13.7)
3–9	262 (50.7)
10 or more	184 (35.6)
Work from home	
Do not work from home	181 (35.0)
One person	230 (44.5)
Two people	105 (20.3)
Three people	1 (0.2)
Data on children	
Sex	
Girls	259 (50.1)
Age (years)	5.3 (2.4)

SD: standard deviation *The minimum wage is equivalent to BRL 1,045.00, the value in force in 2020.

The electronic devices most used by guardians and parents, considering that participants could mention more than one device, were mobile phone and television, which in turn were used less frequently by children. Regarding the daily time spent in front of screens, 64.4% of guardians and parents reported they determine the time by which the child can use electronic devices. The median age at which children were first exposed to any type of electronic device was six months (interquartile range: 1–12 months). The average number of hours that children spent in front of these devices was 3.9±2.3, a value that considers both leisure hours and time to perform school activities. When stratifying the data according to the children’s age, the average hours spent by them in front of screens was different: children between 2 and 5 years of age were on average 3±1.8 hours in front of screens, and those aged between 6 and 9 years, 4.3±2.4 hours (p≤0.001). In addition, 83.9% of the guardians/parents reported that the time spent in front of screens, disregarding the time dedicated to school activities, changed during the pandemic; 81.6% stated that it has increased.

Regarding the guardians/parents, the average hours spent in front of screens to perform work activities and leisure time was 9±2.9. Considering only the time for remote work activities, the average hours spent in front of screens was 6.5±2.9 in this sample. When analyzing the time of screen use of children and guardians/parents, a low correlation was found (r=0.269), although statistically significant. The other variables are described in Table 2.

**Table 2.** Electronic devices used with greater frequency and daily time of exposure to screens of parents or guardians and children aged 2 to 9 years during the COVID-19 pandemic.

	Children (n = 517)	Guardians/Parents (n = 507)	p-value
Most used electronic devices	n (%)	n (%)	0.56
Television	302 (58.4)	427 (82.7)	
Mobile phone	127 (24.6)	509 (98.5)	
Tablet	60 (11.6)	71 (13.7)	
Computer	19 (3.7)	366 (70.8)	
Video game	8 (1.5)	23 (4.4)	
Other (DVD)	1 (0.2)	0 (0)	--
Exposure time to screens (hours)**	3.91 (2.32)	8.96 (2.92)	0.002

*answers with more than 18 hours for work and leisure were excluded, **values expressed in mean (standard deviation).

Concerning food consumption, 60.9% of guardians/parents and 54.3% of children had the habit of eating in front of screens, with a significantly higher prevalence among adults (p<0.001). The most frequently reported meals were lunch, dinner, and breakfast, in the same order for both groups. As for the consumption of snacks outside meal times, when using screens, the prevalence was higher among children compared with their guardians/parents (36.8 and 26.5%, respectively; p<0.001). In these moments, the foods most frequently consumed by children were fruits, home-made sandwiches or snacks, candies and sweets, snacks and salted biscuits. The other variables are described in Table 3.

**Table 3.** Consumption of meals and snacks in front of screens by parents and children aged 2 to 9 years during the COVID-19 pandemic.

	Children n (%)	Guardians/Parents n (%)	p-value
Meals consumed in front of screens			
YES	281 (54.3)	315 (60.9)	
Breakfast	72 (13.9)	107 (20.7)	<0.001
Mid-morning snack	32 (6.2)	23 (4.4)	<0.001
Lunch	178 (34.4)	131 (25.3)	<0.001
Mid-afternoon snack	78 (15.1)	52 (10.1)	<0.001
Dinner	151 (29.2)	122 (23.6)	<0.001
Supper	16 (3.1)	21 (4.1)	<0.001
NO	236 (45.6)	202 (39.1)	
Consumption of snacks in front of screens			
YES	190 (36.8)	137 (26.5)	
Candies and sweets	70 (13.5)	76 (14.7)	<0.001
Soft drinks, chocolate milk, or sugary drinks	23 (4.4)	29 (5.6)	<0.001
Stuffed crackers and ready-made cakes	54 (10.4)	32 (6.2)	0.22
Salted snacks and biscuits	62 (12.0)	41 (7.9)	<0.001
Pizza, nuggets, hamburger, French fries	3 (0.6)	17 (3.3)	0.75
Fruits	134 (25.9)	69 (13.3)	<0.001
Home-made sandwich or snack	88 (17.0)	69 (13.3)	0.002
Vegetables	10 (1.9)	66 (12.8)	0.001
NO	327 (63.2)	380 (73.5)	

The association between the time of exposure to screens and the frequency of consumption of fresh or minimally processed and ultra-processed foods throughout the week was analyzed. There was no significant association between the time of exposure to electronic devices and the frequency of food consumption; however, there was a trend between frequent consumption of hamburgers and processed meats and longer time spent in front of screens by the sample (p=0.059). The other variables are described in Table 4.

**Table 4.** Mean (standard deviation) in hours of exposure time to screens according to the consumption of fresh, minimally processed, and ultra-processed foods by children aged 2 to 9 years during the COVID-19 pandemic.

Consumption of fresh and ultra-processed foods	0-1/per week	2-4/per week	5-7/per week	p-value
Raw salad	4.2 (2.2)	3.9 (2.7)	3.7 (2.8)	0.213
Cooked vegetables	4.1 (2.5)	3.8 (2.2)	3.9 (2.2)	0.517
Fresh fruits or fruit salad	4.0 (2.7)	3.8 (2.0)	3.9 (2.3)	0.926
Beans	3.9 (2.7)	3.8 (2.3)	4.0 (2.2)	0.925
Milk or yogurt	3.9 (2.2)	3.5 (2.0)	4.0 (2.4)	0.439
French fries, potato chips, or fried snacks	3.8 (2.3)	4.2 (2.3)	3.8 (2.9)	0.328
Hamburger or processed meats	3.8 (2.1)	4.0 (2.6)	4.9 (2.8)	0.059
Instant noodles, packet snacks, or salted biscuits.	3.8 (2.2)	4.3 (2.5)	3.6 (2.4)	0.297

## DISCUSSION

The present study aimed to map the time of exposure to screens of families during the COVID-19 pandemic and to associate it with the food consumption of children aged 2 to 9 years. Although broad, the analyzed age group was 2 to 9 years of age, considering that in childhood, more than in other life stages, children do not have full autonomy to make decisions. Thus, they depend on the choices and habits of their guardians/parents to guide aspects related to food consumption as well as those related to daily activities. Children under two years of age were not included because they have specific dietary and nutritional recommendations.

The study data showed a high prevalence of excessive time spent in front of screens during the pandemic. The average time spent using electronic devices was higher in all age groups when compared with the time recommended by the American Academy of Pediatrics for the age.^18^The Brazilian Society of Pediatrics,^17^ in line with the American Academy of Pediatrics,^18^ recommends that the time spent in front of screens should be limited and proportional to the ages and stages of the brain-cognitive-psychosocial development of children. Therefore, it is recommended that preschool children aged between 2 and 5 years use these devices for a maximum of one hour a day, always supervised by guardians/parents, and school children over two years of age should use these devices for up to two hours a day.^17,18^ In addition, it is important that guardians and parents establish limits concerning the type of media consumed and ensure that screen time does not replace adequate sleep, physical exercise, and other behaviors essential to health.^20^

Regarding the pattern of excessive exposure to screens demonstrated by the data analyzed in the present study, the scientific literature has shown similar results for children regardless of age group. A study conducted in the state of Rio Grande do Sul (Brazil), on 658 students aged 7 to 17 years, showed that 55.5% of the sample was exposed to screens for more than two hours a day.^21^ In addition, another Brazilian study, on 1,352 guardians/parents of children aged 0 to 12 years during the COVID-19 pandemic, demonstrated that the time by which children are exposed to screens significantly increased during this period.^9^ Although these studies include the adolescent age group, known to be different in their habits and behaviors when compared with children, no studies that had analyzed screen exposure during the pandemic only focused on children were found.

Furthermore, studies conducted in other countries presented an average exposure to screens higher than that found in the present study.^11^ A recent research conducted by Heller et al. proposes a review of the recommendations for exposure to screen time, as the pandemic period changed the teaching model and this time of exposure for study alone would already extrapolate the current recommendations.^22^ In this sense, when analyzed in a stratified manner, the time of screen use was significantly different between different age groups and, although the average use of screens had been higher than that recommended for both groups, school-age children had a much longer time of screen use, which thereby indicates a time probably dedicated to school activities, as suggested by Heller et al.^22^

Regarding food consumption in front of screens, both children and their guardians/parents had the habit of eating while using electronic devices, a practice that is more frequent among adults. It opposes the recommendations of the *Dietary Guidelines for the Brazilian population*, which is one of the strategies to promote adequate and healthy nutrition for the Brazilian population and presents as one of the recommended aspects for obtaining a healthy eating pattern the act of having meals together. The act of eating calmly and attentively, in appropriate environments and in company whenever possible, influences not only food choices, but also the amount of food ingested by individuals.^23^ In this sense, a study conducted in the United States of America, which analyzed the prevalence of obesity in 8,550 preschool children, showed that children who had the habit of having family dinner at least five times a week, slept 10.5 hours or more per night and watched television for less than two hours a day were 40% less likely to develop obesity when compared with children from families who did not follow these routines.^24^In addition, a study conducted on 1,336 pairs of children-guardians/parents showed that the consumption of fruits, vegetables, and milk was positively associated with the number of days in which the sample dined as a family.^25^

Another practice reported by the parents and guardians interviewed for the present study is the consumption of snacks in front of screens, which, in this case, was more prevalent in children when compared with their guardians/parents. The foods most frequently consumed in these situations were fruits, home-made sandwiches and foods, candies and sweets and, finally, snacks and salted biscuits. It is noteworthy that, with the exception of fruits and dishes made with fresh foods, which are rich in fibers, vitamins, and minerals, the other foods consumed in front of screens are ultra-processed, rich in sugars, fats, and with high calorie density. Other studies corroborate this datum and show that consuming this category of food in front of screens is frequent.^26,27^ Vereecken et al. showed that children who eat while watching television eat more high-fat foods, fast food, pizza, snacks, and soft drinks and fewer fruits and vegetables.^26^ Exposure to screens not only affects aspects related to the time of the meal, as aforementioned, but also exposes children and adolescents to child advertising, usually massive in stimulating the consumption of foods with low nutrient content and high calorie density. This pattern of food consumption in front of screens, if constant, can lead to the development of overweight and obesity in early stages of life.^28,29^ Frutuoso et al. found a significant association between food consumption in front of television and overweight and obesity in adolescents aged 7 to 14 years living in São Paulo (Brazil).^27^

Moreover, in the present study, no association was found between the time by which children were exposed to these devices and the frequency of food consumption throughout the week. However, it was possible to notice a trend in the analyses in the group of hamburgers and processed meats. The longer the time of exposure of children to screens, the higher the frequency of consumption of these foods by the study population, but without statistical significance. Nevertheless, other studies have shown an association between the time of exposure to screens and food consumption.^30,31^ Matheson et al. observed that children consumed less fruit and vegetables when the television was on compared with meals when it was off.^30^ Salmon et al. evaluated 613 children aged between 5 and 6 years and found a significant association between the habit of watching television for two hours or more a day and the consumption of energy drinks and savory snacks as well as an inverse association with the habit of consuming fruits.^31^ It is noteworthy that children’s time of exposure to screens in the present study included both leisure moments and periods of involvement with school activities, without making distinctions. It is likely that food consumption at the time when children are involved in learning activities with teachers and colleagues is not the same as in leisure time.

Although the present investigation did not aim to perform an intrafamilial comparison, the authors observed that, although both guardians/parents and children presented high prevalence in the use of screens and in food consumption in front of them, there were differences. While guardians/parents used the screens mostly for work purposes, children made use of these devices mainly for recreational purposes. Thus, the food pattern in front of screens was also different: adults mostly had the main meals in front of screens, whereas the consumption of snacks was more prevalent among children, probably due to the different use of screen devices. However, once again, it is worth mentioning that this was not the objective of the study and, for any type of additional statement, other analyses with data not collected here would be necessary.

Regarding the limitations of the study, the authors mention the convenience sampling, with data collection performed through an online questionnaire, in which the participant answered without the aid of a researcher. Another limitation of the convenience sampling refers to the extrapolation of data to the Brazilian population. This extrapolation is not possible depending on the sampling format adopted. Nonetheless, online collection enabled to carry out the study during the pandemic. The participants had high level of education, which may have helped in the process of interpreting the questions. Furthermore, we can mention a possible difficulty of parents or guardians in identifying the time during which their children are exposed to screens. This is because many of them tend to consider that the time spent with electronic devices is only that in which the child is actively involved with such devices, excluding periods in which other activities are being carried out with these appliances connected in the same environment. In contrast, the relevance of the investigation of this topic is highlighted mainly during the COVID-19 pandemic, a period in which children had their daily habits widely modified.

The authors conclude that the children analyzed in this study are excessively exposed to screens and that the consumption of meals or snacks in front of these devices is frequent. The use of screens has become necessary for many activities during the pandemic, from school to leisure activities and also as a support tool for parents. In view of the current demands related to the COVID-19 pandemic, the expansion of the use of electronic devices is understandable, but this resource should not impair the time directed to adequate sleep, the act of having meals, family activities, and active playful activities. In this context, the need to educate families in relation to the importance of limiting the use of screens, especially during meals, and to monitor the content of activities involving these devices is reinforced, as exposure to them can influence the quality of food and have repercussions on the nutritional status and health of children in the medium- and long-term. All in all, the expansion of the use of these devices should not extend to later periods, considering its increasingly and widely perceived damage to children’s health and development.

## References

[1] Wu F, Zhao S, Yu B, Chen YM, Wang W, Song ZG (2020). A new coronavirus associated with human respiratory disease in China. Nature..

[2] Brazil - Presidência da República. Lei n. 13.979, de 6 de fevereiro de 2020 (2020). Dispõe sobre as medidas para enfrentamento da emergência de saúde pública de importância internacional decorrente do coronavírus responsável pelo surto de 2019.

[3] Brazil - Ministério da Saúde (2020). Saúde mental e atenção psicossocial na pandemia COVID-19: crianças na pandemia COVID-19.

[4] Borzekowski DL, Robinson TN (2001). The 30-second effect: an experiment revealing the impact of television commercials on food preferences of preschoolers. J Am Diet Assoc..

[5] Coon KA, Tucker KL (2002). Television and children’s consumption patterns. A review of the literature. Minerva Pediatr..

[6] McCurdy LE, Winterbottom KE, Mehta SS, Roberts JR (2010). Using nature and outdoor activity to improve children’s health. Curr Probl Pediatr Adolesc Health Care..

[7] Oliveira JS, Barufaldi LA, Abreu GA, Leal VS, Brunken GS, Vasconcelos SM (2016). ERICA: use of screens and consumption of meals and snacks by Brazilian adolescents. Rev Saúde Pública..

[8] Eyimaya AO, Irmak AY (2021). Relationship between parenting practices and children’s screen time during the COVID-19 pandemic in Turkey. J Pediatr Nurs..

[9] Sá CS, Pombo A, Luz C, Rodrigues LP, Cordovil R (2021). COVID-19 social isolation in Brazil: effects on the physical activity routine of families with children. Rev Paul Pediatr..

[10] Bickham DS, Blood EA, Walls CE, Shrier LA, Rich M (2013). Characteristics of screen media use associated with higher BMI in young adolescents. Pediatrics..

[11] Hare-Bruun H, Nielsen BM, Kristensen PL, Møller NC, Togo P, Heitmann BL (2011). Television viewing, food preferences, and food habits among children: a prospective epidemiological study. BMC Public Health..

[12] Moura NC (2010). Influence of the media upon the feeding behavior of children and adolescents. Segur Alim Nutr..

[13] Bleakley A, Jordan AB, Hennessy M (2013). The Relationship between parents’ and children’s television viewing. Pediatrics..

[14] Birch LL (1999). Development of food preferences. Annu Rev Nutr..

[15] Resnicow K, Davis-Hearn M, Smith M, Baranowski T, Lin LS, Baranowski J (1997). Social-cognitive predictors of fruit and vegetable intake in children. Health Psychol..

[16] Sparrenberger K, Friedrich RR, Schiffner MD, Schuch I, Wagner MB (2015). Ultra-processed food consumption in children from a Basic Health Unit. J Pediatr..

[17] Sociedade Brasileira de Pediatria (2016). Departamento de Adolescência. Saúde de crianças e adolescentes na era digital.

[18] American Academy of Pediatrics (2001). Committee on Public Education. American Academy of Pediatrics: children, adolescents, and television. Pediatrics.

[19] Brasil - Ministério da Saúde (2008). Secretaria de Atenção à Saúde. Departamento de Atenção Básica. Protocols of Feeding and Nutritional Surveillance System – SISVAN in health care (Brazil).

[20] Sociedade Brasileira de Pediatria (2020). Grupo de Trabalho Saúde na Era Digital. Recomendações sobre o uso saudável das telas digitais em tempos de pandemia da COVID-19: boas telas, mais saúde.

[21] Reuter CP, Burgos MS, Pritsch CV, Silva PT, Marques KC, Souza S (2015). Obesity, cardiorespiratory fitness, physical activity and screen time in school children from urban and rural area of Santa Cruz do Sul, Brazil. Cinergis..

[22] Heller NA (2021). Infant media use: a harm reduction approach. Infant Behav Dev..

[23] Brazil - Ministério da Saúde (2014). Secretaria de Atenção à Saúde. Departamento de atenção Básica. Dietary Guidelines for the Brazilian population.

[24] Anderson SE, Whitaker RC (2010). Household routines and obesity in US preschool-aged children. Pediatrics..

[25] Fitzpatrick E, Edmunds LS, Dennison BA (2007). Positive effects of family dinner are undone by television viewing. J Am Diet Assoc..

[26] Vereecken CA, Todd J, Roberts C, Multvihill C, Maes L (2006). Television viewing behavior and associations with food habits in different countries. Public Health Nutr..

[27] Frutuoso MF, Bismarck-Nasr EM, Gambardella AM (2003). Energy expenditure reduction and overweight in adolescents. Rev Nutr..

[28] Leal VS, Lira PI, Menezes RC, Oliveira JS, Costa EC, Andrade SL (2012). Malnutrition and excess weight in children and adolescents: a review of Brazilian studies. Rev Paul Pediatr..

[29] Rezende LF, Lopes MR, Rey-López JP, Matsudo VK, Luiz OC (2014). Sedentary behavior and health outcomes: an overview of systematic reviews. PLoS One..

[30] Matheson DM, Killen JD, Wang Y, Varady A, Robinson TN (2004). Children’s food consumption during television viewing. Am J Clin Nutr..

[31] Salmon J, Campbell KJ, Crawford DA (2006). Television viewing habits associated with obesity risk factors: a survey of Melbourne schoolchildren. Med J Aust..

